# Plasmid-Encoded Nitrogen and Carbon Pathways Enhance Metabolic Flexibility of Multidrug-Resistant Bacteria from Municipal Wastewater

**DOI:** 10.3390/microorganisms14051048

**Published:** 2026-05-07

**Authors:** Shahjahon Begmatov, Andrey L. Rakitin, Yulia Y. Berestovskaya, Alexey V. Beletsky, Andrey V. Mardanov, Nikolai V. Ravin

**Affiliations:** 1 Institute of Bioengineering, Research Center of Biotechnology of the Russian Academy of Sciences, Moscow 119071, Russia; rakitin@biengi.ac.ru (A.L.R.); mortu@yandex.ru (A.V.B.); andrey.mardanov@gmail.com (A.V.M.); 2 Winogradsky Institute of Microbiology, Research Center of Biotechnology of the Russian Academy of Sciences, Moscow 119071, Russia; jberestovskaja@mail.ru

**Keywords:** multidrug-resistant bacteria, wastewater, plasmid, aliphatic amidase, *Klebsiella*, *Pseudomonas*

## Abstract

Wastewater treatment plants represent a primary source of environmental dissemination of multidrug-resistant (MDR) bacteria, underscoring the urgent need for in-depth investigation of these organisms. While the resistome of MDR bacteria has been extensively studied, there remains a critical gap in understanding the role of plasmid-borne genes encoding adaptive metabolic functions. We isolated two MDR strains from municipal wastewater, *Klebsiella* sp. KOS9 and *Pseudomonas veronii* Yu15, both exhibiting resistance to antibiotics, including ampicillin, cefazolin, kanamycin, streptomycin, erythromycin, chloramphenicol, tetracycline, and ciprofloxacin. The plasmids of these strains harbored genes encoding aliphatic amidases, as well as antibiotic resistance genes (ARGs) and enzymes involved in glycogen and dTDP-L-rhamnose biosynthesis, which may contribute to virulence. In *Klebsiella* sp. KOS9 a single acetamidase operon, was found on the megaplasmid, along with copper and silver resistance genes. *P. veronii* Yu15 harbored an operon containing the acetamidase and formamidase genes on the chromosome, as well as a phylogenetically distant acetamidase operon on the conjugative megaplasmid. Both strains exhibit acetamidase activity and *P. veronii* Yu15 was able to utilize acetamide and formamide as sole nitrogen sources. The occurrence of ARGs and adaptive accessory genes on plasmids likely enhances the competitiveness and environmental flexibility of these MDR bacteria.

## 1. Introduction

Microorganisms have evolved diverse mechanisms to outcompete other members of microbial communities, including the production of antibacterial proteins, toxins, and even immune-like systems to suppress rivals [1]. These competitive strategies ultimately serve to secure limited energy resources within their ecological niche. In the gut, microorganisms employ diverse strategies to maintain microbiome stability, such as flexible metabolic pathways, quorum sensing, functional redundancy, and dynamic interactions ranging from cooperation to competition. Crucially, host–microbe interactions further reinforce microbiome equilibrium and ensure its functional integrity [2]. In densely populated microbial niches, such as the gut microbiome or activated sludge at wastewater treatment plants, these adaptive mechanisms undergo continuous evolution, driving the emergence of novel competitive and cooperative strategies. Pathogenic bacteria employ sophisticated survival strategies that enable them to thrive in competitive and hostile environments. Bacterial adaptations drive both virulence (e.g., toxin production) [3] and environmental persistence, with resource availability (such as carbon and nitrogen sources) critically shaping pathogen survival, as seen in *Escherichia coli* [4].

The family *Enterobacteriaceae* comprises clinically significant species alongside well-known genera such as *Salmonella* and *Escherichia*. Notably, *Klebsiella pneumoniae* exhibits remarkable ecological versatility, thriving in diverse niches ranging from free-living environments to host-associated habitats [5]. Molecular epidemiological studies reveal that *K. pneumoniae* sensu stricto and closely related species including *K. quasipneumoniae*, *K. variicola*, *K. quasivariicola*, and *K. africana* vary in their contributions to human disease [6]. The hypervirulent and multidrug-resistant (MDR) strains of *K. pneumoniae* can spread and persist across multiple reservoirs, including humans, animals, wastewater, contaminated water, soil, and medical equipment. Hospital wastewater serves as the primary bacterial reservoir and transmission source. It is subsequently processed at municipal wastewater treatment plants [7].

Bacteria of the genus *Pseudomonas* are ubiquitous in both natural and human-related environments. *Pseudomonas aeruginosa*, a highly adaptable and metabolically versatile species, has been isolated from diverse habitats including the human body, soil, aquatic systems, hydrocarbon-contaminated sites, healthcare facilities, domestic environments and wastewater, where it demonstrates biofilm-forming capacity [8]. Notably, hospital effluents have been shown to contain particularly high concentrations of MDR *P. aeruginosa* isolates [9].

Heterotrophic bacterial pathogens rely on metabolic genes, termed “essential genes”, to exploit available nutrients, including carbon, nitrogen, phosphorus, and sulfur sources, as well as essential micronutrients such as metal ions. These genes encode critical catabolic and anabolic pathways and the regulatory networks governing their expression. Such metabolic systems are well-documented, particularly in model heterotrophic bacteria [10]. These essential metabolic processes are primarily mediated by chromosomally encoded genes. However, plasmids, as extrachromosomal genetic elements, also harbor genes associated with amino acid metabolism, cell wall and capsule biosynthesis, vitamin processing, and utilization of specific carbon and nitrogen sources, highlighting their role in enhancing bacterial metabolic adaptability. For instance, in rhizosphere bacteria, plasmid-encoded nitrogen fixation genes facilitate symbiotic interactions between plants and their bacterial hosts [11]. Additionally, *Lentimonas* bacteria plasmids encode putative carbohydrate-active enzymes (CAZymes), such as fucoidanases, glycoside hydrolases (GHs), sulfatases, and carbohydrate esterases, which play a crucial role in the degradation of recalcitrant polysaccharides [12]. This finding suggests that encoding metabolic properties in plasmids enhances the host organism’s adaptive capacity.

Certain bacterial species possess the metabolic capability to utilize short-chain aliphatic amides as nitrogen sources by enzymatically hydrolyzing these compounds into ammonia and their corresponding organic acids. This reaction is catalyzed by aliphatic amidases (EC 3.5.1.4), a class of enzymes specialized in the cleavage of short-chain amide bonds. Among these enzymes, formamidase AmiF, which catalyzes the hydrolysis of formamide to formate and NH_3_, is a paralog of AmiE amidase, while acetamidase is an orthologous enzyme performing hydrolysis of acetamide (CH_3_CO-NH_2_) to acetic acid and NH_3_ [13]. Acetamidase/formamidase operons have a complex regulatory mechanism, including both positive and negative regulators, and often contain ABC transporter genes [14]. Numerous studies have reported the role of aliphatic amidases in utilizing various amides as nitrogen sources, not only in bacteria but also in fungi [15,16].

As noted above, localization of genes encoding beneficial metabolic functions on plasmids not only expands the adaptive properties of the host organism and confers metabolic flexibility, but also promotes the dissemination of these advantageous traits in microbial populations. A notable example of such beneficial properties is the aliphatic amidase genes carried by plasmids. Plasmids carrying putative amidase genes were revealed by genome sequencing projects, but their functional role remains undocumented.

Here we report the isolation and genome analysis of two MDR strains, *Klebsiella* sp. KOS9 and *Pseudomonas veronii* Yu15, from wastewater treatment plants in Moscow. Both strains exhibited acetamidase activity and contained circular plasmids carrying various advantageous genetic determinants, including ARGs and acetamidase genes. This study represents the first reported finding of plasmid-located aliphatic amidase genes in MDR bacteria. Our findings suggest that plasmid-encoded amidases can enhance the metabolic versatility of bacteria by expanding the range of possible nitrogen sources. This adaptive trait likely contributes to improved bacterial survival and ecological persistence across diverse niches.

## 2. Materials and Methods

### 2.1. Isolation and Characterization of Pure Cultures

Samples of municipal sewage after primary physical treatment, but before biological processing in bioreactors, were collected in August 2024 at the Lyuberetskiy and Kuryanovskiy wastewater treatment plants (WWTP) operated by Mosvodokanal JSC. Serial dilutions of the samples were plated on LB agar simultaneously supplemented with tetracycline (20 µg/mL), chloramphenicol (20 µg/mL), kanamycin (50 µg/mL), and ampicillin (50 µg/mL) as described earlier [17]. In addition, wastewater samples were plated on agarized oligotrophic medium (100 mg/L NH_4_Cl, 20 mg/L MgSO_4_, 20 mg/L NaCl, 70 mg/L KH_2_PO_4_, 245 mg/L K_2_HPO_4_, 100 mg/L yeast extract, 100 mg/L peptone, 250 mg/L glucose), supplemented with the same antibiotics. The plates were incubated at 20 °C for 3–5 days, after which distinct colonies were selected and cultivated in the corresponding liquid medium containing the same antibiotics.

Bacterial cells were harvested by centrifugation at 3000× *g* for 10 min at 4 °C. Genomic DNA was extracted using the DNeasy PowerSoil Pro Kit (Qiagen, Hilden, Germany), and DNA quantification was performed using the Qubit dsDNA HS Assay Kit (Thermo Fisher Scientific, Waltham, MA, USA).

Culture purity was confirmed through microscopic examination of cell morphology and whole-genome sequencing. The 16S rRNA gene was amplified using universal primers 27F and 1492R [18] and sequenced using the Sanger method. Preliminary taxonomic classification was conducted using BLASTN search against the GenBank database. Strain KOS9, isolated on LB and identified as *Klebsiella* sp., and strain Yu15, isolated on oligotrophic medium and identified as *Pseudomonas veronii*, were selected for further study.

To determine the ability of the strains to utilize various nitrogen sources, 1 mL of an overnight culture grown in LB medium supplemented with antibiotics (as described above) was centrifuged to collect cells. The pellet was dissolved in 1 mL of saline, the cells were pelleted again by centrifugation, and the pellet was again dissolved in 1 mL of saline. Bacteria were then streaked onto plates with minimal M9 medium with 3.6 g/L glucose, lacking NH_4_Cl but supplemented with 1 g/L of acetamide, urea, formamide, or NaNO_3_. M9 medium with glucose without NH_4_Cl and with NH_4_Cl was used as negative and positive controls, respectively. To test the ability of the strains to use acetamide and formamide as the sole sources of nitrogen and carbon, the bacteria were streaked on plates with M9 medium without the addition of glucose and lacking NH_4_Cl but supplemented with 1 g/L of acetamide or formamide. The plates were further incubated at 20 °C for 3 days, and the appearance of colonies was recorded.

To determine the susceptibility/resistance of strains KOS9 and Yu15 to antibiotics, bacteria were grown in liquid LB medium overnight at 20 °C and then streaked onto LB plates supplemented with various concentrations of the drugs being studied. The plates were further incubated at 20 °C for 3 days. The minimum inhibitory concentration (MIC) was defined as the lowest concentration that prevented visible bacterial growth. The following antibiotics were used: ampicillin, cefazolin, kanamycin, streptomycin, erythromycin, chloramphenicol, tetracycline, tigecycline, colistin, ciprofloxacin, rifamycin, and fosfomycin.

### 2.2. Genome Sequencing and Analysis

The total genomic DNA of strains *Klebsiella* sp. KOS9 and *P. veronii* Yu15 was sequenced using Illumina and Oxford Nanopore technologies. For Illumina shotgun sequencing, libraries were prepared using the NEBNext Ultra II DNA Library Prep Kit (New England Biolabs, Ipswich, MA, USA) and sequenced on an Illumina MiSeq instrument in a paired-end format (2 × 300 nt). A total of 751,156 read pairs (0.45 Gb) for *Klebsiella* sp. KOS9 and 742,990 read pairs (0.44 Gb) for *P. veronii* Yu15 were generated. Paired-end reads were merged using FLASH v.1.2.11 [19], followed by adapter trimming and low-quality sequence removal (Q < 30) using Cutadapt v.1.8.3 [20] and Sickle v.1.33 (https://github.com/najoshi/sickle acessed on 4 May 2026), respectively. For Nanopore sequencing, genomic DNA was processed using the 1D Genomic DNA Ligation Sequencing Kit (LSK-114) (Oxford Nanopore Technologies, Oxford, UK). *P. veronii* Yu15 DNA was sequenced on a MinION (FLO-MIN111 flow cell) yielding 0.18 Gb (31,456 reads), and FLO-MIN114 flow cell was used for *Klebsiella* sp. KOS9 genome sequencing, producing 0.61 Gb (89,378 reads).

The MinION reads of *P. veronii* Yu15 were assembled using Flye v.2.9.5 [21] with the parameters “—nano-raw” and “—iterations 2”. *Klebsiella* sp. KOS9 MinION reads were assembled with Flye v.2.9.5, and two parameters were specified: “—nano-hq” and “—iterations 2”. The resulting contigs were subsequently polished using Illumina reads via Pypolca v.0.3.1 [22].

The assembled genomes of strains KOS9 and YuLOS-15 were taxonomically classified using the Genome Taxonomy Database Toolkit (GTDB-Tk) v.2.6.1 [23] with reference to the Genome Taxonomy Database (GTDB) [24]. Gene prediction and annotation were performed using the NCBI Prokaryotic Genome Annotation Pipeline [25] and the RAST server 2.0 [26]. Average Nucleotide Identity (ANI) between genomes was calculated using the ani.rb script from the Enveomics collection [27]. Insertion sequence (IS) elements were identified through a BLASTN search (E-value ≤ 1 × 10^−50^) against the ISFinder database [28].

### 2.3. Antibiotic Resistance Gene Identification

Open reading frames (ORFs) were predicted from the assembled contigs using Prodigal v.2.6.3 [29]. ARGs were subsequently identified using the NCBI AMRFinderPlus v.4.0.5 command-line tool [30] with its corresponding database. The predicted protein sequences of all ORFs were analyzed using the parameter -p for comprehensive detection.

### 2.4. Plasmid Sequence Comparison and Visualization

Plasmid sequences were searched against the NCBI RefSeq plasmid database using BLASTN, applying stringent criteria (E-value ≤ 1 × 10^−50^, identity > 80%). From the top ten hits, the best matches with the highest query coverage were selected. Additionally, ANI was calculated for these sequences to assess genomic relatedness. Visualization of the circular plasmid maps and their interactive features was performed using the Proksee expert system [31].

### 2.5. Measurement of Acetamidase Activity

The analyzed strain was grown for 3 days at 30 °C in M9 medium supplemented with glucose (0.1% *w*/*v*), acetamide (0.1% *w*/*v*), and, in the case of *Klebsiella* sp. KOS9, casamino acids (0.2% *w*/*v*). 1.5 mL of the overnight culture (OD_600_ = 1.9) was centrifuged, and the cell pellet was washed once with 1 mL of saline. Then the cells were collected by centrifugation and resuspended in 700 μL of 50 mM Na phosphate buffer (pH = 7.0) supplemented with 1 mg/mL lysozyme [32]. The culture was kept on ice for 30 min and then sonicated five times for 30 s using Bandelin Sonopuls 2200 instrument at 50% power. Insoluble material was removed by centrifugation for 15 min (14,000× *g*) at 4 °C. The total protein concentration in the supernatant was measured using a Qubit Fluorimeter (Thermo Fisher Scientific, Waltham, MA, USA).

An acetamide cleavage reaction was then performed by adding 5–50 µL supernatant (6–8 µg total protein) to 50 mM Na phosphate buffer (pH 7.0) with 0.1 M acetamide (total volume 1 mL). The reaction was run at 30 °C for 30–60 min. As a negative control, the same reaction was performed without the addition of acetamide. The resulting ammonium in was determined by reacting it with Nessler’s reagent to produce a colored complex, the intensity of which was measured spectrophotometrically at OD_450_ and quantified using a calibration curve prepared from known concentrations of NH_4_Cl [33]. The acetamidase activity unit was defined as μM of formed ammonium per minute per 1 μg of total protein.

## 3. Results

### 3.1. Isolation and Genome Sequencing of Two MDR Strains from Wastewater

Bacterial strains designated KOS9 and Yu15 were isolated from municipal wastewater collected at the Kuryanovskiy and Lyuberetskiy WWTPs, respectively. Based on 16S rRNA gene sequencing data, strain KOS9 was assigned to the genus *Klebsiella*, while strain Yu15 was assigned to the genus *Pseudomonas*.

Subsequent sequencing and assembly of the genomes allowed obtaining circular contigs, representing chromosomes and plasmids (Table 1). Taxonomic placement of the KOS9 genome in the GTDB showed that this strain belongs to the candidate species *Klebsiella* sp013705725 found in freshwater sample collected downstream of wastewater treatment plant in UK (*Klebsiella* sp. RHBSTW-00484 in NCBI). ANI value between the KOS9 genome and this genome (GCA_013705725.1) was 99.4%, which confirms the assignment of KOS9 strain to this candidate species.

To further characterize the taxonomic status of the KOS9 strain, a maximum likelihood phylogenetic tree based on 120 conserved genes was constructed for the KOS9 genome and the genomes of all other validly described *Klebsiella* species from GTDB (Figure S1). The closest relative of KOS9 was *Klebsiella huaxiensis* WCHKl090001 (GCF_003261575.2) with an ANI of 94.81%, which is slightly below the 95% threshold for species delimitation [34]. Therefore, strains KOS9 and RHBSTW-00484 likely represent a distinct species of *Klebsiella* that has yet to be formally described.

The second strain, Yu15, was assigned to the species *Pseudomonas veronii* based on 98.9% ANI to *P. veronii* strain R02 (GCF_001439695.1).

Genome assembly revealed that both strains possess a circular chromosome along with eight plasmids in KOS9 and seven plasmids in Yu15 (Table 1). Plasmid sizes ranged from 1240 to 150,051 bp for KOS9 and from 2736 to 226,692 bp for Yu15. In strain KOS9, plasmids pKOS9-W4 and pKOS9-W3 encode conjugation proteins of the IncF and IncI1 groups of plasmids, respectively. Furthermore, plasmid pKOS9-W1 contains an incomplete set of IncF-plasmid conjugation genes (*traT*, *traD*, *traI*, *traX*, and *finO*). In the Yu15 strain, clusters of genes encoding IncP-type and IncF-type conjugative transfer proteins were found on plasmids pYu-LOS15-16 and pYu-LOS15-17, respectively, while plasmid pYu-LOS15-12 carried the *trb/virB* operon promoting conjugal transfer.

**Figure 1:**
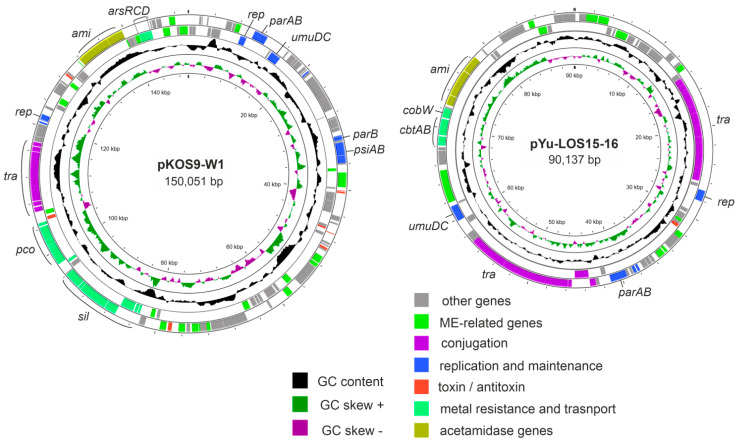
Schematic maps of plasmids pKOS9-W1 and pYu-LOS15-16. Protein-coding genes on the forward and reverse strands are shown as rectangles outside and inside the main circle, respectively. The innermost circle presents GC-skew in green (+) and purple (−), and the next-to-innermost circle represents G + C content (deviation from the average) in black (+, outward; −, inward). ME, mobile element.

Plasmid pKOS9-W1 contained a 21-kb locus containing genes conferring resistance to copper (*pcoABCDRSE*) and silver (*silRSE/silCFBAP*) (Figure 1). This plasmid also harbored the arsenate reductase gene *arsC*, together with the genes *arsR* and *arsD.* Plasmid pKOS9-W2 contains the mercury resistance operon (*merRTPCADE*). A tellurium resistance gene cluster (*ter*), comprising 17 genes, was found on plasmid pKOS9-W4 along with the mercury resistance operon. On the contrary, metal resistance genes were not found on the plasmids of strain Yu15.

### 3.2. Resistomes of Klebsiella sp. KOS9 and P. veronii Yu15

The results of ARG prediction revealed that the chromosome of *Klebsiella* sp. KOS9 contains four ARGs, while its plasmids carry 17 (Table S1). The chromosomal genes *aph(3′)-I*, *gyrA_T83I*, and *bla*_*OXY*-12-__1_ could confer resistance to kanamycin, quinolones, and cephalosporins, while the *emrD* gene encoding an efflux pump could confer resistance to various drugs. All 17 plasmid-borne ARGs were located on the conjugative plasmid pKOS9-W4 and include genes associated with resistance to β-lactams (*bla_TEM_*, *bla*_*DHA*-1_, *bla*_*SHV*-12_), aminoglycosides (*aac(3)-IIg*, *aac(6′)-IIc*, *aph(3″)-Ib*, *aph(6)-Id*, *aac(6′)-Ib*), tetracyclines (*tet(D)*), sulfonamide (two *sul1* genes), phenicols (*catA2*), trimethoprim (*dfrA19*), quinolones (*qnrB*), macrolides (*ereA*), rifamycin (*arr*), and colistin (*mcr-9.1*). The largest (~18 kb) resistance gene island contains five directly oriented copies of the IS26 element and ARGs located between them (*tet(D)*, *aac(6′)-Ib*, *bla*_*SHV*-12_, and *catA2*). Besides ARGs this cluster contains genes involved in the L-threonate degradation pathway: 3-oxo-tetronate kinase, L-threonate dehydrogenase, and a transcriptional regulator of the DeoR family. Such loci, bounded by directly oriented transposons of the IS26 family, are pseudo-compound transposons that can move together as a coherent single unit [35]. Another long ARG locus was associated with class 1 integron, widely known for its role in the dissemination of antibiotic resistance [36]. It contained the genes *aac(6′)-IIc*, *ere(A)*, *aac(3)-IIg*, *arr*, *qacE*, and *sul1*. The *ere(A)* gene seems to be broken by an insertion of IS1247 element linked to the *aac(3)-IIg* and *arr* genes. Another class 1 integron comprised the genes *aph(3″)-Ib* and *aph(6)-Id*.

The chromosome of *P. veronii* Yu15 contained seven ARGs (*aph(6)-Id*, *aph(3″)-Ib*, *cmx*, *sul1*, *ere(A)*, *ampC*, *fos*) (Table S1). Five of them were linked to class 1 integron, while *ampC* and *fos* were located distantly. Very close homologs of integron-linked ARGs (99–100% nucleotide sequence identity) were found in *P. aeruginosa*, but in other strains of *P. veronii* they are missing. All other 10 ARGs (*aadA1*, *bla*_*OXA*-10_, *qnrVC1*, *aph(3′)-Ia*, *tmexC3*, *tmexD3*, *toprJ*, *sul1*, *floR2*, *tet(G)*) were located on the largest conjugative plasmid pYu-LOS15-17 (Table S1). This plasmid is closely related (more than 99% nucleotide sequence identity over 75% of its length) to the pHN39-SIM plasmid from clinical strain *Pseudomonas aeruginosa* HN39 (KU254577). Other strains of *P. veronii* lacked plasmids similar to pYu-LOS15-17 (homologous regions covered less than 11% of its length). ARGs carried by pYu-LOS15-17 can confer resistance to aminoglycosides, β-lactams, quinolones, tetracyclines, sulfonamides, and phenicols. All plasmid-borne ARGs were located in a single 32-kb region flanked by two direct copies of the IS6100 element. This region contained two class 1 integrons and a pseudo-compound transposon consisting of two direct copies of the IS26 element and the *aph(3′)-Ia* gene between them. IS6100-flanked ARG cassettes were also identified in the 27.8-kb R-plasmid pTET3 from *Corynebacterium glutamicum* [37]. Of particular interest is the *tmexC3D3-toprJ* gene cluster found within this region, encoding the RND family multidrug efflux pump. This cluster is associated with resistance to several classes of antibiotics and is widespread among clinically relevant bacterial pathogens, including *Pseudomonas* spp. *Klebsiella pneumoniae* strains carrying such cluster have been increasingly detected in clinical, veterinary, and food samples in China. The presence of this cluster contributes to multidrug resistance, notably to tigecycline, one of the last-resort antimicrobial agents used to treat infections caused by multidrug-resistant *Enterobacteriaceae* [38,39]. A BLASTN search revealed multiple close homologs of fragments of this 32-kb region in plasmids of *Pseudomonas aeruginosa*, but none had an identical genetic structure.

The detected sets of ARGs explain the phenotypic resistance of strains KOS9 and Yu15 to tetracycline, ampicillin, kanamycin, and chloramphenicol which were used for their selective isolation. Results of additional antibiotic susceptibility testing showed that both strains also exhibited resistance to cefazolin, streptomycin, erythromycin, and ciprofloxacin (Table 2). *Klebsiella* sp. KOS9 was also resistant to colistin and rifamycin, while resistance to fosfomycin was observed for *P. veronii* Yu15. The observed susceptibility/resistance profiles are consistent with the ARG sets in both strains (Table 2). Although the *ere(A)* gene appears to be nonfunctional in strain KOS9, its resistance to erythromycin may be mediated by the multidrug efflux transporter *emrD*.

### 3.3. Genes Encoding Aliphatic Amidases

Analysis of *Klebsiella* sp. KOS9 and *P. veronii* Yu15 genomes revealed the presence of aliphatic amidase genes in both strains. To experimentally evaluate acetaminidase activity, the strains were grown in LB medium with the addition of 0.1% acetamide to activate transcription of the acetaminidase operon [40]. Acetaminidase activity was detected in cell lysates, amounting to 3355 U/mg in strain KOS9 and 3989 U/mg in strain Yu15.

**Figure 2:**
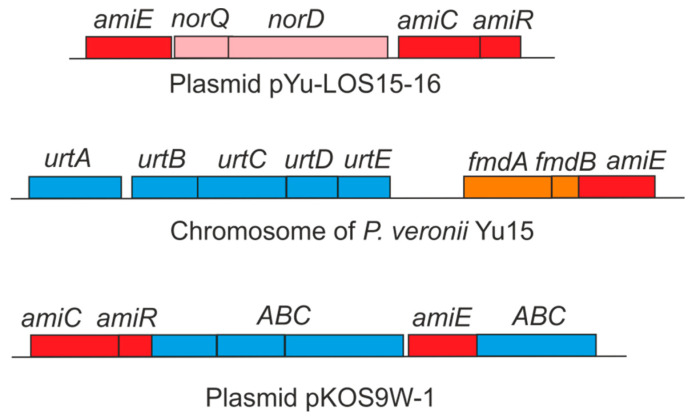
Clusters of genes comprising acetaminidase operons. Rectangles immediately above the main line represent predicted genes; their colors indicate functional classification as follows: acetaminidase (red), formamidase (orange), accessory genes in the *ami* operon (pink), ABC-type transporter subunits (blue). All genes are oriented rightward.

In the KOS9 strain, a single acetaminidase gene, *amiE*, was identified on the pKOS9W-1 plasmid (Figure 1). The AmiE enzyme (EC 3.5.1.4) catalyzes the hydrolysis of acetamide to produce ammonia, which can be utilized as a nitrogen source for growth. This *amiE* gene is part of an operon that also includes genes *amiC*, *amiR*, and four genes encoding subunits of the ABC-type transporter (Figure 2). The periplasmic binding domain of AmiC serves as the ligand sensor and negative regulator of the acetamidase operon [14]. AmiR acts as the RNA-binding positive regulator enabling transcription antitermination. According to previous reports, under non-inducing or repressing growth conditions, AmiC and AmiR form a complex that silences the activity of AmiR [41]. The encoded ABC transporter system belongs to cluster 5, includes two permease proteins, a substrate-binding protein, and an ATP-binding protein, and probably enables the uptake of acetamide into the cell. The 8 kb acetamidase operon, analyzed via BLASTN search, exhibited high sequence similarity (>99%) to various plasmids from the *Enterobacteriaceae*, including *Raoultella*, *Phytobacter*, *Citrobacter*, *Klebsiella*, *Enterobacter*, etc.

Two acetaminidase operons were found in the Yu15 strain, located on the chromosome and on the pYu-LOS15-16 plasmid (Figure 1). The chromosomal cluster contained the aliphatic amidase gene *amiE*, the *fmdA* gene encoding formamidase, the *fmdB* gene encoding a positive regulator of formamidase, and the *urtABCDE* genes for the urea ABC transporter (Figure 2). Genes *amiC* and *amiR* were not found in this locus. Colocalization of the *urtABCDE* and formamidase genes suggested that this transporter could actually be involved in the uptake of formamide. Very close homologs of this cluster (>99% nucleotide sequence identity) were found in chromosomes of different *Pseudomonas* species, including *Pseudomonas veronii* OST1911.

The plasmid operon, in addition to the *amiE*, *amiC*, and *amiR* genes, includes two more genes (Figure 2). The first one encodes a protein containing the ATPase AAA domain PFAM07728 fused to the PFAM08406 domain found at the C-terminus of proteins of the CbbQ/NirQ/NorQ family of proteins which can act as activators of nitrite and nitric oxide reductases. The second gene encoded NorD-like protein, a molecular chaperone for the maturation of nitric oxide reductase. These proteins could be involved in the assembly/activation of aliphatic amidase, although their roles remain unclear. Plasmid and chromosomal *amiE* genes are dissimilar at the nucleotide level (74% identity over 83% of the length) and a GenBank search using BLASTN revealed homologues of the whole plasmid *ami* operon (70–75% identity) in the chromosomes of *Hydrogenophaga* sp. PAMC20947 and several other betaproteobacteria of the order *Burkholderiales*, but not in *Pseudomonas* sp. The *ami* operon was likely acquired by the pYu-LOS15-16 plasmid through horizontal transfer from a phylogenetically distant species.

Since the Yu15 strain could grow on minimal medium (M9) with glucose as a carbon source, we tested its ability to utilize acetamide and formamide as sole nitrogen sources. The strain grew successfully on plates with M9 medium and glucose lacking NH_4_Cl but supplemented with 1 g/L of acetamide or formamide. Moreover, this strain is able to grow on M9 medium without both glucose and NH_4_Cl, but with acetamide or formamide, suggesting that these compounds can be used as sole sources of both nitrogen and carbon.

The inability of the KOS9 strain to grow on minimal medium prevented us from testing its ability to use acetamide as a nitrogen and/or carbon source.

### 3.4. Utilization of Other Nitrogen Sources

Since the primary function of aliphatic amidase is to provide the bacterium with nitrogen, which is important under conditions of ammonium deficiency in the environment, we analyzed the genomes of strains KOS9 and Yu15 for the presence of genes that could enable the utilization of other nitrogen sources.

Both strains have chromosomal genes for urease (*ureABC*) and its accessory proteins, as well as the urea ABC transporter (*urtABCDE*). The genome of strain Yu15 additionally contains genes for urea carboxylase and allophanate hydrolase. These enzymes can hydrolyze urea to form ammonia either in a single-step reaction (urease) or with the formation of urea-1-carboxylate as an intermediate. Strain Yu15 was able to grow on M9 medium with glucose, lacking NH_4_Cl but supplemented with 1 g/L of urea, thus confirming its ability to utilize it as a sole nitrogen source.

Ethanolamine can serve as another source of nitrogen. Ethanolamine is abundant in the human gut, and the ability of gut bacteria to use it as nitrogen and/or carbon source often aids their survival in inflamed host intestines [42]. Both strains contain operons including ethanolamine permease and ethanolamine ammonia-lyase, which breaks down ethanolamine to form acetaldehyde and NH_3_. In the Yu15 strain, this operon also includes the gene for NAD^+^-dependent acetaldehyde dehydrogenase, which catalyzes the irreversible oxidation of acetaldehyde into acetic acid. The chromosome of the KOS9 strain additionally contains an *eut* operon comprising 17 genes, including ones encoding structural proteins of the bacterial microcompartment (BMC), an organelle bound by a proteinaceous shell and used to metabolize toxic compounds. In *Salmonella enterica* the BMC encapsulates enzymes that generate and degrade acetaldehyde, formed by ethanolamine ammonia-lyase [43]. Acetaldehyde is a volatile and reactive metabolite and could escape as a gas; therefore its sequestration and metabolization in BMC also increase the efficiency of utilization of ethanolamine as a carbon source.

Both strains possess nitrate and nitrite transporters, as well as assimilatory nitrate reductase and NAD(P)H-nitrite reductase (NirBD), which catalyze the reduction of nitrate and nitrite to ammonium. The ability of strain Yu15 to assimilate nitrate and use it as a nitrogen source was confirmed by its ability to grow on minimal medium supplemented with 1 g/L of NaNO_3_ as a sole nitrogen source.

In addition to nitrate reductase NasA, strain KOS9 also has respiratory nitrate reductase (NarGYI), which enables utilization of nitrate as an electron acceptor under anaerobic conditions. Strain Yu15, in addition to NarGYI, possesses dissimilatory cytochrome *cd1* nitrate reductase NirS, nitric oxide reductase, and nitrous oxide reductase. This dissimilatory denitrification pathway was encoded in a single chromosomal locus.

### 3.5. Plasmid Genes Mediating Carbon Metabolism

The plasmids of strain KOS9, in addition to genes involved in acetamide utilization, contain gene clusters associated with carbohydrate metabolism. Plasmid pKOS9-W3 contains a gene cluster for the biosynthesis of dTDP-L-rhamnose from deoxythymidine triphosphate (dTTP) and glucose-1-phosphate [44]. dTDP-L-rhamnose is an important precursor of O-antigen, a major component of the surface lipopolysaccharide in Gram-negative bacteria. This locus includes genes encoding glycosyltransferase, an O-antigen export system (ATP-binding protein and permease protein), dTDP-4-dehydrorhamnose 3,5-epimerase (EC 5.1.3.13), dTDP-4-dehydrorhamnose reductase (EC 1.1.1.133), glucose-1-phosphate thymidylyltransferase (EC 2.7.7.24), and dTDP-glucose 4,6-dehydratase (EC 4.2.1.46). This 7 kb long region is present in various *Enterobacteriaceae*, with the closest homolog found in the pIncX3 plasmid (CP036195.1) of the *Klebsiella pneumoniae* strain BA34918. Interestingly, these genes were also found in the chromosome of strain KOS9 where they are included in a larger biosynthetic gene cluster. However, the nucleotide sequences of the chromosomal and plasmid copies of these genes were dissimilar, indicating that the plasmid genes originated from other bacterial species.

Additionally, the pKOS9-W2 plasmid contains a cluster of genes involved in glycogen biosynthesis, including a 1,4-alpha-glucan (glycogen) branching enzyme (GH-13-type, EC 2.4.1.18), glucose-1-phosphate adenylyltransferase (EC 2.7.7.27), glycogen synthase (ADP-glucose transglucosylase, EC 2.4.1.21), glycogen phosphorylase (EC 2.4.1.1), and phosphoglucomutase (EC 5.4.2.2). This genomic region exhibits hundreds of homologs in the NCBI database, and is commonly present in plasmids from *Klebsiella*, *Raoultella*, *Enterobacter*, and *Escherichia* species. Gene cluster with the same gene order and content is also present in the chromosome; the nucleotide sequence identity between the chromosomal and plasmid copies of these genes was above 90%.

## 4. Discussion

### 4.1. ARGs and Their Origin

Most pathogenic bacteria, such as *P. aeruginosa*, can grow in a minimal medium with a single carbon and energy source and are capable of metabolizing numerous organic compounds [45]. This metabolic versatility enhances their persistence in diverse ecological niches and enables their survival within the host microenvironment [46]. Certainly, virulence factors and antibiotic resistance mechanisms enable these pathogenic bacteria to survive in the host and outcompete other members of the microbial community. Recent studies have shown that MDR bacteria employ diverse mechanisms, facilitating their colonization of an expanding range of ecological niches. In this process, they naturally broaden their spectrum of substrate utilization for energy acquisition.

MDR strains of *Klebsiella* were isolated from wastewater worldwide. For instance, in Shanghai, 13 strains of the *K. pneumoniae* species complex were identified among 3574 wastewater treatment plant samples. Most of these isolates exhibited resistance to carbapenems and colistin, along with hypervirulence [47]. Similarly, *K. pneumoniae*, the most frequently detected species, was isolated from hospital wastewater and clinical specimens in a Brazilian hospital. These isolates carried *Klebsiella pneumoniae* carbapenemase (KPC) or New Delhi metallo-β-lactamase (NDM), demonstrating their persistence in hospital wastewater [48]. Numerous studies have documented the isolation and identification of MDR *Klebsiella* from wastewater [49,50]. The clinical origin of such strains is well-established, as exemplified by the frequent isolation of *Klebsiella* spp. from blood samples in Moscow hospitals. Genomic analyses of these strains revealed the presence of resistance genes to major classes of antibiotics [51].

Interestingly, all 17 extrachromosomal ARGs in the KOS9 strain were located on a single megaplasmid, pKOS9-W4. Bacterial plasmids also often contain genes associated with resistance to metals and other toxic compounds. Plasmid pKOS9-W4 carried arsenic and mercury resistance operons, as well as a tellurium resistance gene cluster. The *ter* gene cluster, besides tellurium detoxification, enhances bacterial survival against oxidative stress, phagocytosis, phages, and colicins. It is often found on plasmids of pathogenic bacteria and is strongly associated with virulence through the elimination of oxidative damage, increasing fitness and resistance to reactive oxygen species during macrophage attack [52]. The presence of a complete set of IncF-type conjugative transfer genes suggests that the pKOS9-W4 plasmid can be transmitted by conjugation and disseminate ARGs along with other resistance and virulence determinants in bacterial populations.

*P. veronii* was initially isolated from natural mineral waters [53] and was later found in different aquatic systems and soils. It is known for its metabolic flexibility and ability to degrade organic pollutants such as aromatic hydrocarbons, including naphthalene, toluene, and n-alkanes [54,55,56]. The genome of the *P. veronii* strain Yu15 also contained numerous genes associated with the degradation of these compounds (alkanes, benzoate, etc.). Clinical and/or pathogenic strains of *P. veronii* have not yet been described, and antibiotic resistance has not been reported for cultured isolates of this species. However, a metagenome-assembled genome assigned to *P. veronii* was recently obtained from mastitis-affected cow’s milk [57]. This MAG contained several ARGs, mostly encoding multidrug efflux pumps [57].

Strain *P. veronii* YuLOS-15 is the first MDR member of this species, containing 17 ARG conferring resistance to most important classes of antibiotics. All non-chromosomal ARGs (10 in total) were located on a single IncF-type conjugative megaplasmid (pYu-LOS15-17), very similar to plasmids of the known pathogen *P. aeruginosa*, but not to plasmids of other strains of *P. veronii*. It is possible that the *P. veronii* Yu15 strain acquired this plasmid from clinical strains of *P. aeruginosa* via conjugative transfer. Similarly, the chromosomal locus containing five AGRs has very close homologs in *P. aeruginosa* genomes but not in other strains of *P. veronii.* Therefore, acquisition of chromosomal ARGs by the ancestor of *P. veronii* Yu15 from *P. aeruginosa* via horizontal gene transfer is a likely scenario.

### 4.2. Plasmid-Carried Genes Involved in dTDP-L-rhamnose Biosynthesis and Glycogen Metabolism

Plasmids in both strains contain genes for the biosynthesis of dTDP-L-rhamnose from glucose-1-phosphate and dTTP. The key genes in this pathway, dTDP-4-dehydrorhamnose-3,5-epimerase, dTDP-4-dehydrorhamnose reductase, and dTDP-glucose-4,6-dehydratase, have homologs on plasmids in the NCBI database (accessed on 19 June 2025). These plasmids are predominantly from *Alphaproteobacteria* (582, 499, and 509 entries, respectively), with *Sinorhizobium* being the most frequently represented genus. dTDP-L-rhamnose serves as a precursor of L-rhamnose, a saccharide critical for the virulence of some pathogenic bacteria. Because this metabolic pathway is absent in humans, the enzymes involved represent potential targets for new antimicrobials. In Gram-negative bacteria such as *Salmonella enterica*, *Vibrio cholerae*, and *Escherichia coli* O75:K53, L-rhamnose is a key component of the O-antigen in lipopolysaccharides, which are essential for serum resistance and host colonization. In Gram-positive bacteria, including streptococci, L-rhamnose promotes capsule formation, a well-known virulence factor [58]. The frequent presence of these genes in *Sinorhizobium* may be related to their symbiotic lifestyle, which involves host colonization. Similarly, *Klebsiella* sp. KOS9 and *P. veronii* Yu15 strains may use similar plasmid-encoded mechanisms to enhance virulence.

The survival of *Klebsiella* sp. KOS9 can be enhanced by glycogen biosynthesis genes located on the pKOS9W-2 plasmid. Glycogen primarily functions as a key energy reserve in prokaryotes, and recent studies indicated its critical role in tolerance to extreme environmental conditions, host colonization, and virulence. For example, inhibitors targeting the GlgB protein have been shown to effectively reduce the virulence and pathogenicity of *Mycobacterium tuberculosis* [59]. Furthermore, deletion of *glgB* and *glgX* in *Escherichia coli* resulted in increased biofilm biomass and decreased viability under stressful conditions, highlighting their importance for bacterial virulence and survival in harsh environments [60]. The presence and functionality of the glycogen metabolism gene cluster in the KOS9 plasmid pKOS9W-2 may contribute to the adaptability of this strain.

### 4.3. Aliphatic Amidases and Their Functional Roles

Previous studies have shown that bacteria can utilize aliphatic amides as a source of nitrogen and/or carbon. Amidases involved in this metabolic pathway can hydrolyze short-chain aliphatic amides, such as formamide, acetamide, propionamide, butyramide, valeramide, acrylamide, and nitrilamide, to the corresponding carboxylic acids and ammonia [61].

According to the NCBI database (accessed on 25 February 2026), 112,956 bacterial proteins were annotated as acetamidases or aliphatic amidases. Of these, only 1777 acetamidases and 383 aliphatic amidases were encoded by genes located on plasmids. It is interesting that in *Klebsiella*, acetamidase genes were found on plasmids much more often: 1040 and 4869 genes on plasmids and chromosomes, respectively. Plasmids appear to be common carriers of aceamidase genes in *Klebsiella* sp., enabling recipient bacteria to utilize acetamide as a source of nitrogen, and possibly carbon as well. This is precisely what was observed in the KOS9-9 strain, whose only acetaminidase gene is localized on a plasmid rather than a chromosome.

Genetic loci completely identical to the acetamidase gene cluster from the pKOS9-W1 plasmid of *Klebsiella* sp. KOS9 were found on plasmids of *Phytobacter diazotrophicus* and *Klebsiella (Raoultella) ornithinolytica*, a species of *Enterobacteraleceae*. Initially identified as a plant growth-promoting bacterium [62], *P. diazotrophicus* has recently been recognized as an opportunistic pathogen associated with nosocomial infections in clinical settings. Notably, *P. diazotrophicus* has been reported to harbor plasmids containing the *bla_NDM_* gene, potentially facilitating the spread of carbapenem resistance [62]. *K. ornithinolytica* was commonly found in water and soil, but is currently being considered as an emerging pathogen causing human infections [63]. Since this *ami* gene cluster is the only one in the *Klebsiella* sp. KOS9 genome, it is the pKOS9-W1 plasmid that confers the strain the ability to hydrolyze acetamide. This plasmid also contains copper and silver resistance gene clusters, which further contribute to the strain’s adaptive capabilities.

The frequencies of occurrence of aliphatic amidases genes on chromosomes and plasmids in *Pseudomonas* sp. differ significantly from those in *Klebsiella* sp. Only three aliphatic amidase and acetamidase genes were found on plasmids, in contrast to the 2124 genes found on *Pseudomonas* chromosomes. The genome of the *P. veronii* strain Yu15 contained two aliphatic amidase gene clusters. The first, including the acetamidase and formamidase genes, as well as the ABC transporter, was located on the chromosome and showed high similarity to chromosomal loci in various *Pseudomonas* species. The second cluster, located on the pYu-LOS15-17 plasmid, was apparently acquired by lateral transfer from an unknown beta-proteobacterium. In accordance with predictions based on genome analysis, both strains indeed exhibit enzymatic activity against acetamide.

The primary role of the aliphatic amidase genes in *Klebsiella* sp. KOS9 and *P. veronii* Yu15 may be used to provide these bacteria with additional nitrogen and, possibly, carbon sources. Aliphatic amidases can exhibit activity on several related substrates. For example, AmiE from *Helicobacter pylori* efficiently hydrolyzes acetamide, acrylamide, and propionamide, but not formamide, while AmiF specifically hydrolyzes formamide. Although acetamide occurs naturally in limited quantities, primarily in red beet roots, it is often used as an industrial solvent, plasticizer, and can be found in industrial wastewater. Formamide is a common industrial wastewater pollutant originating from chemical, pharmaceutical, and textile manufacturing.

In the *Klebsiella* strain KOS9, a single acetamidase gene was found on the pKOS9-W1 plasmid, the presence of which could confer adaptive advantages on the host. In the *P. veronii* strain Yu15, the presence of an additional plasmid-carried acetamidase operon, distantly related to the chromosomal *ami* operon, may have allowed for the utilization of a broader range of short-chain aliphatic amides. The ability to utilize a wide range of nitrogen sources seems to be important for both strains, as indicated by the presence of corresponding metabolic pathways in the genomes. In addition to aliphatic amidases, both strains possess genetic mechanisms for urea and ethanolamine utilization, as well as assimilatory nitrate reduction. Moreover, *P. veronii* Yu15 can also use acetamide and formamide as carbon sources, which is consistent with data obtained for other *Pseudomonas* species [64].

In addition to its primary metabolic role in providing the host bacterium with the ability to utilize additional nitrogen and/or carbon sources, acetamidase activity may indirectly influence virulence of *Pseudomonas aeruginosa* [65]. The eukaryotic C-type natriuretic peptide hormone CNP binds to the *P. aeruginosa* AmiC sensor, inducing *amiE* transcription. Overexpression of *amiE* resulted in reduced biofilm formation and overproduction of rhamnolipids. Furthermore, AmiE influenced swarming, twitching, and production of the quorum-sensing molecules, overall causing a decrease in virulence [65]. The possible role of aliphatic amidase genes in *Klebsiella* sp. KOS9 and *P. veronii* Yu15 beyond utilization of aliphatic amides remains to be explored.

## 5. Conclusions

The results of this study represent the first report on the resistome and mobilome of multidrug-resistant *Klebsiella* sp. KOS9 and *P. veronii* Yu15 strains isolated from municipal wastewater. Numerous ARGs were identified on the plasmids, many of which were associated with mobile elements. The strains also contained clusters of aliphatic amidase genes, enabling the utilization of short-chain amides as carbon and nitrogen sources. The inventory of beneficial genes carried by the plasmids suggests an important role of plasmids in the persistence of these MDR bacteria in adverse environmental conditions and, potentially, in their virulence.

## Figures and Tables

**Table 1:** Genome characteristics of *Klebsiella* sp. KOS9 and *P. veronii* Yu15.

Replicon *	Length (bp)	GC Content	Copy Number **	Protein-Coding Genes	Acetamidase/Formamidase Genes	Other Features of Plasmids	ARGs
*Klebsiella* sp. KOS9							
Chromosome	6,309,390	53.13	(1)	5694			4
pKOS9-W1	150,051	52.13	1.9	143	*amiCRE*	*pco*, *sil*	
pKOS9-W2	95,453	53.81	2.8	97		*mer*	
pKOS9-W3	73,930	49.71	4.4	91		IncI1-type conjugative	
pKOS9-W4	350,575	47.22	1.7	357		IncF-type conjugative, *ter*, *mer*	17
pKOS9-W5	6408	42.51	6.5	9			
pKOS9-W6	4841	48.71	15.7	5			
pKOS9-W7	1240	46.13	7.8	1			
pKOS9-W8	2964	45.82	18.7	4			
*P. veronii* Yu15							
Chromosome	7,035,211	60.54	(1)	5759	*amiE*, *fmdAB*		7
pYu-LOS15-09	2736	60.71	8.5	3			
pYu-LOS15-10	4841	54.61	3.6	8			
pYu-LOS15-11	9192	48.46	1.4	12			
pYu-LOS15-12	30,686	53.42	1.4	31		conjugative, *trb/virB* operon	
pYu-LOS15-15	66,106	52.79	1.7	47			
pYu-LOS15-16	90,137	55.16	1.8	87	*amiECR*	IncP-type conjugative	
pYu-LOS15-17	226,692	56.03	1.7	209		IncF-type conjugative	11

* chromosome and plasmids; ** relative to the chromosome according to average sequence coverage.

**Table 2:** MIC values of antibiotics for *Klebsiella* sp. KOS9 and *P. veronii* Yu15.

Class of Antibiotics	Antibiotic	MIC (mg/L) for KOS9	MIC (mg/L) for Yu15
Beta-lactam	Ampicillin	>2000	>2000
	Cefazolin	>200	>200
Aminoglycoside	Kanamycin	>2000	>2000
	Streptomycin	>500	>500
Macrolide	Erythromycin	2000	>2000
Phenicol	Chloramphenicol	1000	500
Tetracycline	Tetracycline	>1000	500
	Tigecycline	20	5
Polymyxin	Colistin	160	20
Quinolone	Ciprofloxacin	60	30
Rifamycin	Rifamycin	100	<1
Fosfomycin	Fosfomycin	50	250

## Data Availability

The complete genome sequences (chromosomes and plasmids) of *Klebsiella* sp. KOS9 and *P. veronii* Yu15 have been deposited in the NCBI GenBank database under the accession numbers CP200238-CP200246 and JBXEEG010000001-JBXEEG010000008, respectively.

## Supplementary Materials

The following supporting information can be downloaded at: https://www.mdpi.com/article/10.3390/microorganisms14051048/s1, Figure S1: Genome-based analysis of phylogenetic position of *Klebsiella* sp. KOS9 in the genus *Klebsiella*; Table S1: ARGs detected in the genomes of *Klebsiella* sp. KOS9 and *P. veronii* Yu15.
