# Supplementary material for: Plasmid-Encoded Nitrogen and Carbon Pathways Enhance Metabolic Flexibility of Multidrug-Resistant Bacteria from Municipal Wastewater
Source: Microorganisms. 2026 May 7;14(5):1048. doi: 10.3390/microorganisms14051048 (PMC13209695; doi:10.3390/microorganisms14051048)
Supplement: Supplementary file 1 [file microorganisms-14-01048-s001.zip › Figure S1.pdf]

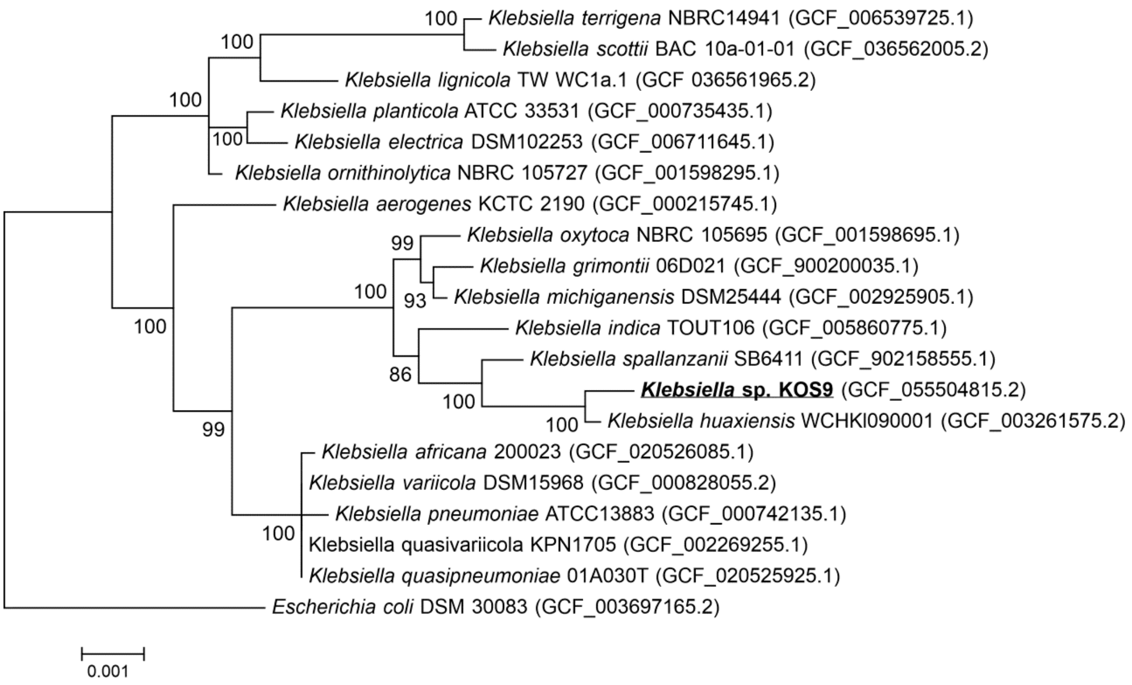

Figure S1. Genome-based analysis of phylogenetic position of *Klebsiella* sp. KOS9 in the genus *Klebsiella*.

GTDB-Tk v.2.6.1 was used to identify 120 single-copy marker genes in the genomes and to construct a concatenated multiple amino acid sequence alignment from the KOS9 genome and reference *Klebsiella* genomes retrieved from GenBank. A phylogenetic tree was inferred using PhyML v.3.3 with default parameters; branch support was assessed using the Bayesian test implemented in PhyML. GenBank assembly accession numbers are shown after the genome names.
