# Supplementary material for: Plasmid-Encoded Nitrogen and Carbon Pathways Enhance Metabolic Flexibility of Multidrug-Resistant Bacteria from Municipal Wastewater
Source: Microorganisms. 2026 May 7;14(5):1048. doi: 10.3390/microorganisms14051048 (PMC13209695; doi:10.3390/microorganisms14051048)
Supplement: Supplementary file 1 [file microorganisms-14-01048-s001.zip › Table S1.pdf]

Supplemental Table S1. ARGs detected in the genomes of *Klebsiella* sp. KOS9 and *P. veronii* Yu15.

Location	Gene	Product	Drug class	Drug subclass
***Klebsiella* sp. KOS9**				
chromosome	*aph(3')-I*	APH(3')-I family aminoglycoside O-phosphotransferase	aminoglycoside	gentamicin/ kanamycin
chromosome	*gyrA_T83I*	Klebsiella oxytoca quinolone resistant GyrA	quinolone	quinolone
chromosome	*bla_{OXY-12-1}*	extended-spectrum class A beta-lactamase OXY-12-1	beta-lactam	cephalosporin
chromosome	*emrD*	multidrug efflux MFS transporter EmrD	multiple	multiple
pKOS9-W4	*bla_{TEM}*	TEM family class A beta-lactamase	beta-lactam	beta-lactam
pKOS9-W4	*dfrA19*	trimethoprim-resistant dihydrofolate reductase DfrA19	trimethoprim	trimethoprim
pKOS9-W4	*aph(3'')-Ib*	aminoglycoside O-phosphotransferase APH(3'')-Ib	aminoglycoside	streptomycin
pKOS9-W4	*aph(6)-Id*	aminoglycoside O-phosphotransferase APH(6)-Id	aminoglycoside	streptomycin
pKOS9-W4	*mcr-9.1*	phosphoethanolamine-lipid A transferase MCR-9.1	colistin	colistin
pKOS9-W4	*tet(D)*	tetracycline efflux MFS transporter Tet(D)	tetracycline	tetracycline
pKOS9-W4	*aac(6')-Ib*	AAC(6')-Ib family aminoglycoside 6'-N-acetyltransferase	aminoglycoside	amikacin/ kanamycin/ tobramycin
pKOS9-W4	*bla_{SHV-12}*	extended-spectrum class A beta-lactamase SHV-12	beta-lactam	cefiderocol/ cephalosporin
pKOS9-W4	*catA2*	type A-2 chloramphenicol O-acetyltransferase CatII	phenicol	chloramphenicol
pKOS9-W4	*sul1*	sulfonamide-resistant dihydropteroate synthase Sul1	sulfonamide	sulfonamide
pKOS9-W4	*bla_{DHA-1}*	extended-spectrum class C beta-lactamase DHA-1	beta-lactam	cephalosporin
pKOS9-W4	*qnrB*	QnrB family quinolone resistance pentapeptide repeat protein	quinolone	quinolone
pKOS9-W4	*sul1*	sulfonamide-resistant dihydropteroate synthase Sul1	sulfonamide	sulfonamide
pKOS9-W4	*ere(A)**	EreA family erythromycin esterase	macrolide	erythromycin
pKOS9-W4	*arr*	NAD(+)--rifampin ADP-ribosyltransferase	rifamycin	rifamycin
pKOS9-W4	*aac(3)-IIg*	aminoglycoside N-acetyltransferase AAC(3)-IIg	aminoglycoside	gentamicin
pKOS9-W4	*aac(6')-IIc*	aminoglycoside N-acetyltransferase AAC(6')-IIc	aminoglycoside	gentamicin/ kanamycin/ tobramycin

***Pseudomonas veronii* Yu15**				
chromosome	*aph(6)-Id*	aminoglycoside O-phosphotransferase APH(6)-Id	aminoglycoside	streptomycin
chromosome	*aph(3")-Ib*	aminoglycoside O-phosphotransferase APH(3")-Ib	aminoglycoside	streptomycin
chromosome	*cmx*	chloramphenicol efflux MFS transporter Cmx	phenicol	chloramphenicol
chromosome	*sul1*	sulfonamide-resistant dihydropteroate synthase Sul1	sulfonamide	sulfonamide
chromosome	*ere(A)*	EreA family erythromycin esterase	macrolide	erythromycin
chromosome	*ampC*	class C beta-lactamase	beta-lactam	beta-lactam
chromosome	*fos*	fosfomycin resistance glutathione transferase	fosfomycin	fosfomycin
pYu-LOS15-17	*aadA1*	ANT(3")-Ia family aminoglycoside nucleotidyltransferase AadA1	aminoglycoside	streptomycin
pYu-LOS15-17	*bla*$_{OXA-10}$	oxacillin-hydrolyzing class D beta-lactamase OXA-10	beta-lactam	cephalosporin
pYu-LOS15-17	*aac(6')-Ib*	AAC(6')-Ib family aminoglycoside 6'-N-acetyltransferase	aminoglycoside	amikacin/ kanamycin/ tobramycin
pYu-LOS15-17	*qnrVC1*	quinolone resistance pentapeptide repeat protein QnrVC1	quinolone	quinolone
pYu-LOS15-17	*aph(3')-Ia*	aminoglycoside O-phosphotransferase APH(3')-Ia	aminoglycoside	kanamycin
pYu-LOS15-17	*tmexC3*	multidrug efflux RND transporter periplasmic adaptor subunit TMexC3	tetracycline	tigecycline
pYu-LOS15-17	*tmexD3*	multidrug efflux RND transporter permease subunit TMexD3	tetracycline	tigecycline
pYu-LOS15-17	*toprJ*	multidrug efflux transporter outer membrane subunit TOprJ	tetracycline	tigecycline
pYu-LOS15-17	*sul1*	sulfonamide-resistant dihydropteroate synthase Sul1	sulfonamide	sulfonamide
pYu-LOS15-17	*floR2*	chloramphenicol/florfenicol efflux MFS transporter FloR2	phenicol	chloramphenicol/ florfenicol
pYu-LOS15-17	*tet(G)*	tetracycline efflux MFS transporter Tet(G)	tetracycline	tetracycline

* the *ere(A)* in pKOS9-W4 is broken by the insertion of a mobile element and is likely nonfunctional.
